# Cold stress adaptation in *Trifolium ambiguum*: physiological and transcriptomic insights

**DOI:** 10.3389/fpls.2025.1645123

**Published:** 2025-10-16

**Authors:** Kefan Cao, Huimin Zhang, Yiming Ma, Fan Huang

**Affiliations:** 1 Institute of Grassland Research, Chinese Academy of Agricultural Science, Hohhot, China; 2 College of Grassland Science/Key Laboratory of Grassland Resources of Ministry of Education, Inner Mongolia Agricultural University, Hohhot, China

**Keywords:** *Trifolium ambiguum* M. Bieb, cold stress, antioxidant enzymes, osmotic adjustment, RNA-seq

## Abstract

**Introduction:**

Cold stress significantly affects the growth and distribution of leguminous forage crops. *Trifolium ambiguum*, a cold-tolerant perennial clover, exhibits distinct physiological and molecular responses to cold stress. Understanding its cold adaptation mechanisms is crucial for improving the resilience of forage crops.

**Methods:**

In this study, we analyzed the physiological and transcriptomic responses of *T. ambiguum* seedlings exposed to 0°C for 0, 2, 6, and 12 hours. Various physiological parameters, including antioxidant enzyme activities (SOD, POD, and CAT), proline, and soluble sugar levels, were measured. Transcriptomic analysis was performed to identify the gene expression changes associated with cold stress.

**Results:**

The results indicated that antioxidant enzyme activities (SOD, POD, and CAT) significantly increased early in response to cold stress, while proline and soluble sugar levels gradually accumulated, suggesting oxidative defense and osmotic adjustment. Transcriptomic analysis revealed that, during the early stage of cold stress, genes involved in antioxidant metabolism and photosynthesis were upregulated. As the stress persisted, metabolic processes were suppressed, followed by the activation of genes related to flavonoid biosynthesis in the later stages, which helped enhance stress tolerance.

**Discussion:**

These findings provide insights into the cold adaptation mechanisms of *T. ambiguum*. The early upregulation of antioxidant and photosynthesis-related genes, along with the activation of flavonoid biosynthesis pathways later on, suggests a complex and staged response to cold stress. These insights could inform the breeding of cold-resistant forage crops, contributing to more resilient leguminous species in colder climates.

## 1 Introduction

*Trifolium ambiguum* M. Bieb., commonly known as Caucasian clover, is a perennial leguminous forage plant characterized by strong cold resistance, drought tolerance (Hay et al., 2010), and excellent regenerative ability (Townsend, 1970; Bryant, 1974). Due to its high nutritional value and environmental adaptability, *T. ambiguum* has attracted attention for its application in high-altitude and cold regions (Taylor and Smith, 1998). Recent studies have highlighted its potential for enhancing soil fertility and preventing desertification in arid and semi-arid regions (Han et al., 2025). However, the molecular mechanisms underlying its superior cold tolerance remain largely unclear.

Cold stress is a major environmental factor affecting plant growth, development, and geographic distribution (Xu et al., 2020; Kong, 2024). Plants respond to low-temperature stress through a series of morphological, physiological, and molecular changes, including membrane lipid remodeling, accumulation of compatible solutes, and activation of stress-responsive gene expression (Zhao et al., 2019; Wang et al., 2021; Lu et al., 2023). In recent years, transcriptome sequencing (RNA-seq) has become a powerful tool for studying global gene expression profiles in plants under abiotic stress (Zhou et al., 2023a). Several studies have identified cold-responsive transcription factors (e.g., CBFs, bZIPs, MYBs), signaling kinases, and functional genes involved in osmoprotection and ROS scavenging pathways in model plants and crops (Xie et al., 2022; Zhou et al., 2023a).

Despite progress in other legumes, the cold response mechanisms of *T. ambiguum* have not been systematically investigated (Xu et al., 2020). Previous reports have mainly focused on its morphological or agronomic performance under low temperatures, while the underlying regulatory networks at the transcriptomic level are still unknown (Xu et al., 2023).

In addition to transcriptomic and metabolomic studies, plant physiological responses, including the accumulation of osmoprotectants (e.g., proline, soluble sugars, and soluble proteins) and the activation of antioxidant enzymes such as superoxide dismutase (SOD), peroxidase (POD), and catalase (CAT), play a crucial role in maintaining cellular homeostasis and mitigating oxidative damage under cold stress (Zhang et al., 2023). Therefore, integrating physiological indicators with multi-omics data can provide a more comprehensive understanding of plant cold tolerance mechanisms.

In this study, we performed dynamic transcriptome analysis of *T. ambiguum* seedlings subjected to 4°C for different durations (0 h, 2 h, 6 h, and 12 h), combined with physiological parameter measurements and qRT-PCR validation. The aims were to identify key genes and pathways involved in the cold stress response and to provide theoretical insights into the molecular regulatory network underlying cold tolerance in *T. ambiguum*.

## 2 Materials and method

### 2.1 Experimental materials and seed treatment

This study utilized *T. ambiguum* seeds, provided by Inner Mongolia Agricultural University (registration number: N010), as the experimental material. The experimental site is located in Hohhot, Inner Mongolia, at an altitude of 1,000 meters above sea level, with a latitude of 40.8183° N and longitude of 111.7465° E. To ensure uniform germination and experimental reproducibility, the seeds underwent the following standardized treatments: 1) Seed cleaning: *T. ambiguum* seeds were thoroughly rinsed with distilled water to remove impurities, ensuring they were free of contaminants. 2) Seed priming: The cleaned seeds were wrapped in moist filter paper and placed in petri dishes to maintain moisture. The seeds were incubated in the dark at 25°C for 12 hours to promote uniform germination. 3) Germination cultivation: The primed seeds were transferred to a growth medium containing 1/2 Hoagland’s solution and placed in a Percival growth chamber at a constant temperature of 4°C. The seeds were exposed to a 16-hour photoperiod (70% light intensity) and 8 hours of darkness for seven days. 4) Seedling transplantation: After 7 days, the germinated seedlings were transplanted into 10 cm × 10 cm containers filled with a substrate mixture of vermiculite and Pindstrup soil in a 1:1 ratio. The seedlings were cultivated under the same light/dark cycle and environmental conditions for an additional 7 days to support growth and development.

### 2.2 Cold stress treatment

Fourteen-day-old *T. ambiguum* seedlings were randomly divided into treatment and control groups. The experimental design was as follows: 1) Treatment group: Seedlings were exposed to a 4°C environment to simulate cold stress, with samples collected at 2, 6, and 12 hours post-treatment. This temperature was chosen to simulate mild cold stress, a common experimental condition for cold tolerance studies in legumes (Gao et al., 2024b). 2) Control group: Seedlings were maintained at 25°C under standard growth conditions, without cold treatment.

Each group consisted of three biological replicates. Following the treatments, leaf samples were immediately collected, flash-frozen in liquid nitrogen, and stored at −80°C for subsequent molecular and physiological analyses. The sample labels were designated as follows: control group (CK), cold treatment for 2 hours (H2), cold treatment for 6 hours (H6), and cold treatment for 12 hours (H12).

### 2.3 Physiological index determination

Physiological indicators were measured to evaluate the biochemical responses of *T. ambiguum* to cold stress. Leaf samples collected at 0 h (CK), 2 h, 6 h, and 12 h of 0°C treatment were used for biochemical assays. Soluble protein (SP), malondialdehyde (MDA), soluble sugars (SS), catalase (CAT), peroxidase (POD), superoxide dismutase (SOD), and proline (Pro) contents were determined using commercially available detection kits (Solarbio, Beijing, China) according to the manufacturer’s protocols.

Enzyme activities were expressed as U g^-^¹ FW (fresh weight).

SP content was determined using the Coomassie Brilliant Blue G-250 method. MDA content was analyzed based on the thiobarbituric acid (TBA) reaction. SS content was measured using the anthrone colorimetric method. Proline was extracted and quantified according to the ninhydrin reaction method. Activities of CAT, POD, and SOD were determined using ultraviolet spectrophotometry. Three biological replicates were used for each treatment.

### 2.4 Comprehensive transcriptome sequencing and data analysis workflow

The RNA extraction was performed using the RNAprep Pure Plant Kit (Tiangen, Beijing, China) for plants and TRIzol Reagent (Life Technologies, California, USA) for animals, followed by RNA quantification and integrity assessment using NanoDrop 2000 and the RNA Nano 6000 Assay Kit on the Agilent Bioanalyzer 2100 system. For transcriptome sequencing, 1 μg of RNA per sample was used to prepare sequencing libraries with the Hieff NGS Ultima Dual-mode mRNA Library Prep Kit, involving mRNA purification, cDNA synthesis, end-repair, adaptor ligation, PCR amplification, and quality assessment on the Agilent Bioanalyzer 2100 system. Libraries were sequenced on an Illumina NovaSeq platform to generate 150 bp paired-end reads.

Raw reads were processed to obtain clean data through quality control steps, including adapter removal and low-quality read filtering. Clean reads were aligned to the *T. ambiguum* reference genome (version 3.0) using HISAT2 (version 2.2.1). Gene expression levels were quantified using FPKM (Fragments Per Kilobase of transcript per Million mapped reads), while differential expression analysis was conducted with DESeq2 (version 1.34.0) for biological replicates or edgeR (version 3.32.1) for non-replicates, with thresholds set at an adjusted *P*-value < 0.01 and Fold Change ≥ 2. KEGG pathway enrichment analysis was performed using KOBAS (version 3.0) and clusterProfiler (version 3.18.0) to identify significantly enriched pathways. Additionally, alternative splicing events were quantified using rMATS software (version 4.0.2).

The time points of 2, 6, and 12 hours were selected based on preliminary experiments showing significant physiological changes at these intervals. At the 2-hour time point, an immediate response to cold stress is expected, which includes the activation of antioxidant and stress-responsive genes. The 6-hour time point represents an intermediate stage, where metabolic adjustments such as energy redistribution and secondary metabolism are likely to occur. At the 12-hour time point, more established adaptive mechanisms are expected, including the accumulation of osmolytes and activation of genes involved in membrane stabilization and protection. These time points were chosen to capture key phases of the cold stress response in *T. ambiguum*.

This workflow ensured the generation of high-quality transcriptomic data, providing a robust foundation for comprehensive molecular analyses. Sample-to-sample Pearson correlation coefficients were calculated in R software (version 4.3.1) using the cor() function, and the full correlation matrix is provided in **Supplementary Table 1**.

### 2.5 qRT-PCR validation

To validate the results of the differentially expressed genes (DEGs), 15 DEGs were randomly selected for measurement using qRT-PCR. Primers were designed using Pri-mer Premier 5.0 software (**Supplementary Table 2**). The qRT-PCR was performed following the instructions provided in the TB Green Premix Ex Taq™ II kit (Takara).

The reaction system included 10 μL of TB Green Premix Ex Taq™ II, 1 μL of for-ward primer, 1 μL of reverse primer, 1 μL of cDNA, and 7 μL of RNase-Free H_2_O, for a total volume of 20 μL. The relative expression levels of genes were calculated using the 2^-△△Ct^ method. All gene expression analyses were performed with three biological replicates.

## 3 Results

### 3.1 Changes in physiological indices of *T. ambiguum* under cold stress

To further investigate the physiological response mechanisms of *T. ambiguum* under cold stress, key physiological indices, including soluble protein (SP), malondialdehyde (MDA), soluble sugars (SS), catalase (CAT), proline (Pro), peroxidase (POD), and superoxide dismutase (SOD), were measured and analyzed across different cold treatment durations.

SP content showed a significant increase at 2 hours of cold stress (146.05 ± 0.70 μg g^-^¹) compared to the control (141.41 ± 1.67 μg g^-^¹, *p* < 0.05), suggesting that *T. ambiguum* rapidly accumulates functional proteins in response to the initial environmental stress. This early increase in SP likely reflects the activation of protective mechanisms, including the stabilization of cellular structures and enzymes, to counteract the stress-induced damage. MDA content peaked at 6 hours (38.54 ± 1.07 μmol g^-^¹), significantly higher than the control (31.97 ± 0.51 μmol g^-^¹), indicating increased lipid peroxidation and oxidative damage to cell membranes during the mid-phase of stress. This suggests that, during prolonged cold exposure, *T. ambiguum* experiences heightened oxidative stress, which may be linked to the accumulation of reactive oxygen species (ROS). SS content continuously increased with prolonged cold treatment, reaching 16.82 ± 0.60 μg g^-^¹ at 12 hours, more than three times the level in the control (5.31 ± 0.18 μg g^-^¹). This significant accumulation of SS highlights its critical role as an osmoprotectant in maintaining cellular turgor and preventing dehydration under cold stress, which is essential for cellular homeostasis. CAT activity was significantly elevated at 2 hours (91.07 ± 1.74 U g^-^¹), but gradually declined over time, reaching the lowest level at 12 hours (26.82 ± 1.47 U g^-^¹). This temporal pattern suggests that CAT is activated early in the cold stress response to scavenge hydrogen peroxide, but its efficiency declines with prolonged stress, possibly due to enzyme inactivation or depletion of antioxidants. The decline in CAT activity, despite the ongoing stress, may indicate a shift in the plant’s antioxidant defense mechanisms toward other enzymes, such as POD and SOD. Proline content exhibited a consistent upward trend throughout the treatment, peaking at 25.65 ± 0.75 μg g^-^¹ at 12 hours, approximately 2.6-fold higher than the control. Proline is known to play a dual role in osmotic adjustment and ROS scavenging. Its accumulation in the later stages of cold stress likely reflects its role in maintaining cellular turgor and protecting cellular structures from oxidative damage, particularly when other antioxidant defenses begin to decline. POD activity was significantly increased at both 2 hours (31,347.33 ± 1,131.22 U g^-^¹) and 6 hours (30,262.00 ± 72.11 U g^-^¹), suggesting its active role in the antioxidant defense system during the early and middle stages of cold stress. In contrast, SOD activity surged to 228.94 ± 0.88 U g^-^¹ at 2 hours, 2.4 times higher than the control, reflecting its key role in detoxifying superoxide radicals. However, SOD activity gradually declined to 159.18 ± 4.15 U g^-^¹ at 12 hours, indicating a shift in the dominant antioxidative mechanism as stress persisted.

**Figure 1:**
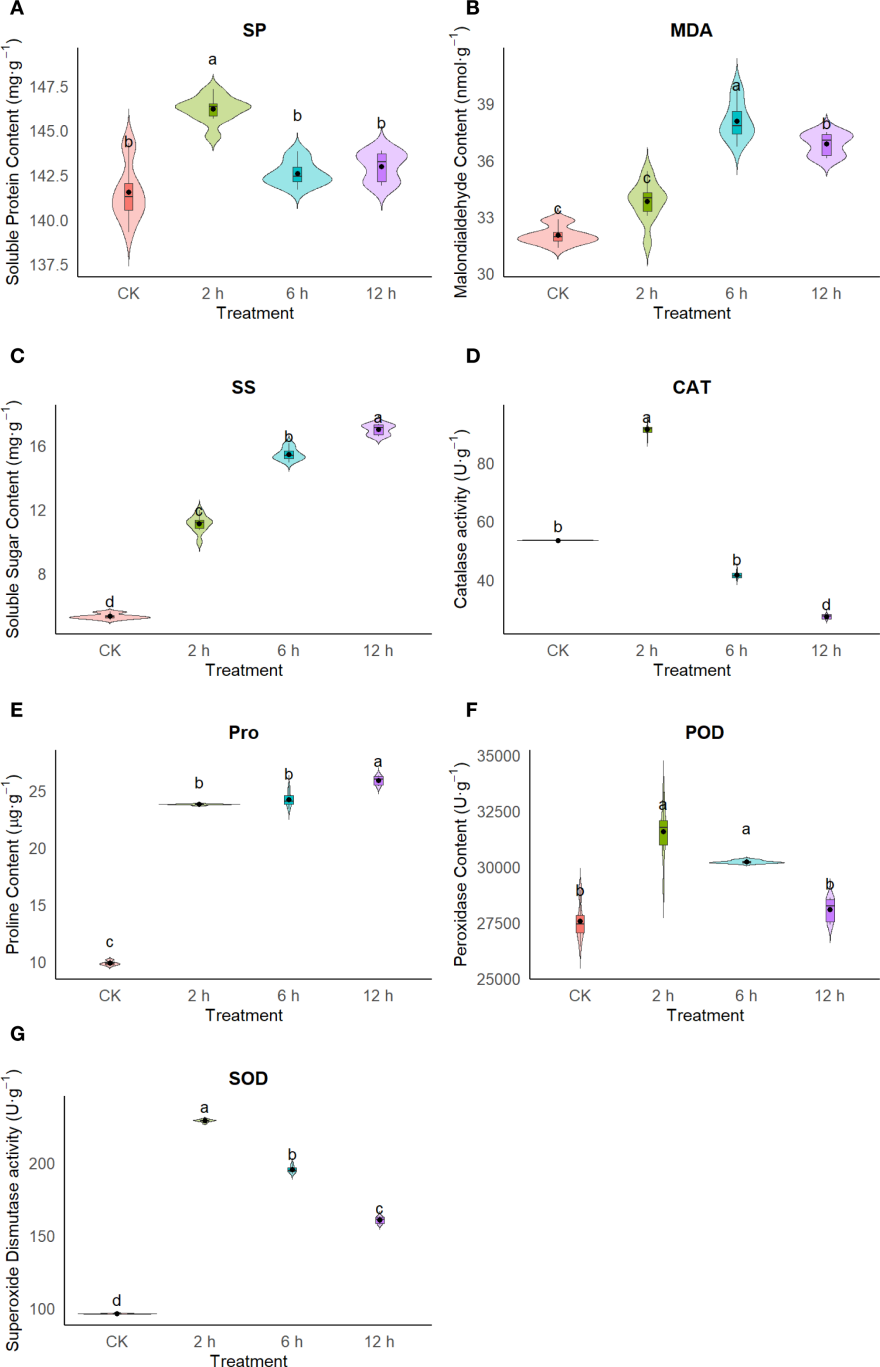
Changes in physiological indices of *T. ambiguum* under cold stress at different time points (0 h, 2 h, 6 h, and 12 h). **(A)** Soluble protein (SP); **(B)** malondialdehyde (MDA); **(C)** soluble sugars (SS); **(D)** catalase (CAT); **(E)** proline (Pro); **(F)** peroxidase (POD); **(G)** superoxide dismutase (SOD). Values are means ± SD (n = 3). Different letters indicate statistically significant differences (*p* < 0.05).

Collectively, the dynamic changes in these physiological indices suggest that *T. ambiguum* maintains cellular homeostasis under cold stress through mechanisms such as osmotic adjustment, antioxidant defense, and protective protein accumulation. Notably, the coordinated regulation of SOD, POD, CAT, and Pro contributes significantly to oxidative stress mitigation, while the accumulation of SS and SP provides metabolic and osmotic protection. These physiological responses are highly consistent with the transcriptomic data, which revealed enrichment of pathways related to antioxidant metabolism, osmotic regulation, and secondary metabolism, providing physiological evidence for the molecular mechanisms underlying cold tolerance (**Figure 1**).

### 3.2 Quality assessment of transcriptome sequencing

The quality assessment of *T. ambiguum* transcriptome sequencing data under cold stress demonstrated that the total read counts (ReadSum) per sample ranged from 20.23 million to 23.76 million, with total base counts (BaseSum) between 605 million and 711 million, indicating sufficient sequencing depth and excellent consistency across samples. The GC content (GC%) ranged from 41.25% to 41.75%, aligning with the genomic characteristics of *T. ambiguum*, with no significant deviations observed. The N content (N%) remained consistently low at 0.01%, indicating minimal contamination. Quality metrics revealed Q20 values above 99% and Q30 values exceeding 97%, reflecting high sequencing accuracy suitable for differential gene expression analysis and functional annotation.

Biological replicates from the treatment groups (CK, H2, H6, H12) exhibited consistent performance across all quality metrics, particularly the stability of GC content and sequencing quality scores, underscoring the rigor of the experimental design and the reliability of data collection. Overall, the data exhibited high sequencing depth, quality, and consistency, providing a robust foundation for investigating gene expression changes and regulatory mechanisms under cold stress (**Supplementary Table 3**).

### 3.3 Gene expression profiles and sample clustering analysis of *T. ambiguum* under cold stress conditions

We utilized RNA-seq technology to examine transcriptomic changes in *T. ambiguum* leaves under cold stress and further explored the molecular mechanisms underlying its transcriptomic response by integrating prior research. Four treatment conditions were included in the study: the control group (CK) and cold stress treatments for 2 hours (H2), 6 hours (H6), and 12 hours (H12). A total of 12 RNA-seq samples were generated, with three biological replicates per group. Alignment results showed successful mapping of all samples to the *T. ambiguum* reference genome, with alignment rates ranging from 73.87% to 77.94%, demonstrating high data quality and reliability.

**Figure 2:**
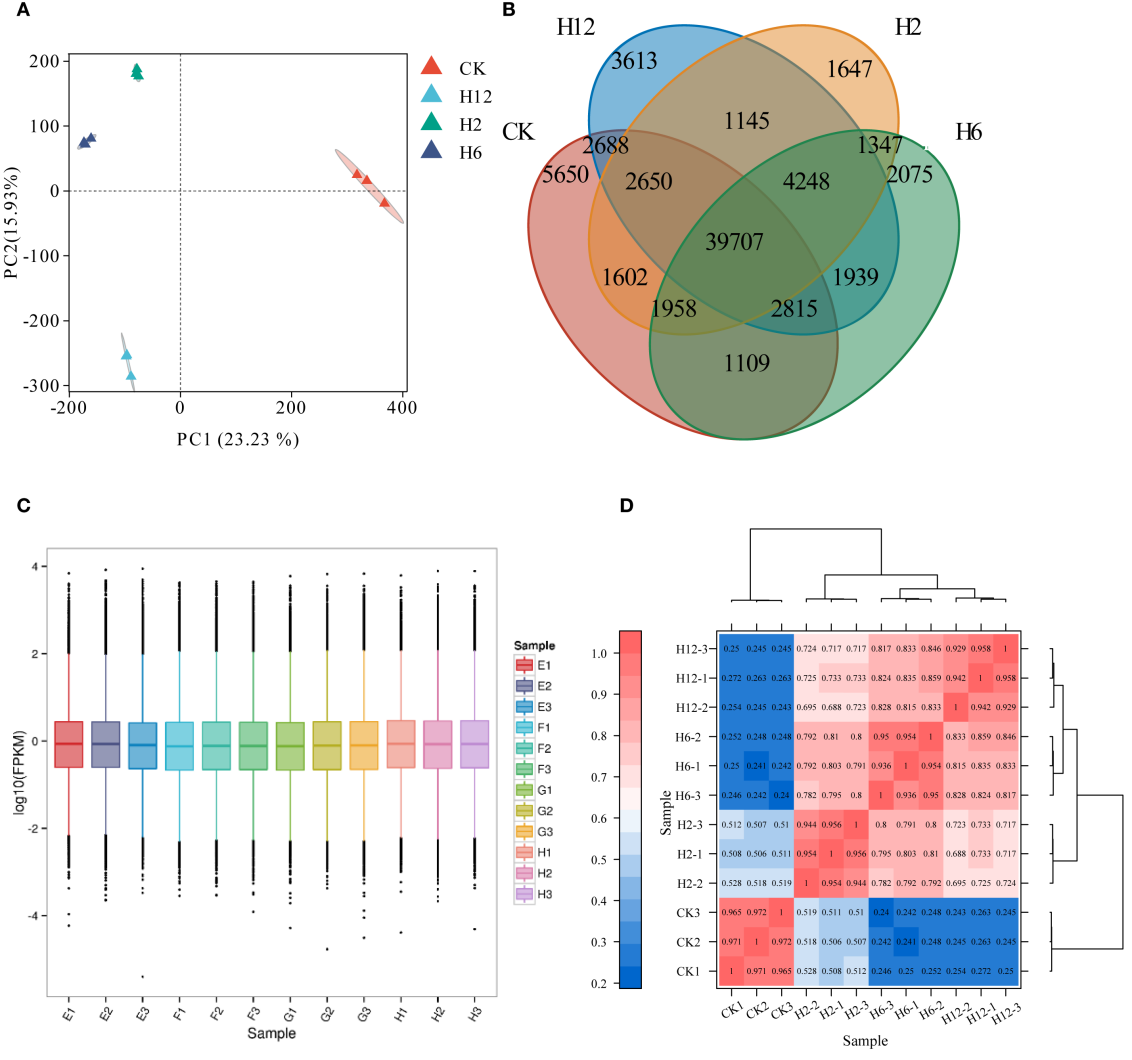
Transcriptomic analysis of *T. ambiguum* under cold stress conditions. **(A)** Principal Component Analysis (PCA) of transcriptome data from *T. ambiguum* under cold stress conditions. **(B)** Venn diagram showing the overlap of differentially expressed genes (DEGs) across various cold stress conditions. **(C)** Boxplot representing the distribution of gene expression levels (log10(FPKM)) for all RNA-seq samples. **(D)** Heatmap of Pearson correlation coefficients across all RNA-seq samples to illustrate the consistency and clustering of biological replicates under different cold stress conditions.

Gene expression correlations (FPKM values) among biological replicates were calculated, yielding an average Pearson correlation coefficient (PCC) greater than 0.98, with a minimum value of 0.96, indicating excellent consistency between samples. Principal Component Analysis (PCA) further confirmed distinct expression patterns between the experimental groups. The first principal component (PC1) accounted for 23.23% of the total variance, clearly separating the control group from the cold stress groups, confirming that cold stress was the primary factor influencing gene expression. The second principal component (PC2) explained 15.93% of the variance, highlighting trends in gene expression changes across different cold stress time points. The tight clustering of biological replicates indicated high data consistency and minimal batch effects (**Figure 2A**).

Venn diagram analysis revealed the distribution of gene expression across the four groups, identifying 39,707 genes commonly expressed under all conditions, underscoring their essential roles in basic metabolism and cell function maintenance. The number of specific genes increased with stress duration, from 1,647 in H2 to 3,613 in H12, reflecting the dynamic impact of stress on gene regulation. The significant rise in specific genes in the H12 group suggests their involvement in adaptation mechanisms to prolonged cold stress. Additionally, 4,248 genes were co-expressed in the H2, H6, and H12 groups under cold stress, likely playing critical roles in core metabolic pathways for cold adaptation. Significant differences in gene expression were observed between treatment groups, with fewer specific genes in H2 and H6, and a pronounced increase in H12, indicating that prolonged stress elicited a more complex regulatory network (**Figure 2B**).

Boxplot analysis of gene expression demonstrated consistent distribution and similar median values across all samples, verifying data stability and reliability (**Figure 2C**). Heatmap clustering analysis revealed significant expression differences between the control and cold stress groups, with higher correlation observed among the cold stress groups (H2, H6, H12). This pattern suggests a conserved stress response, with time-dependent regulation (**Figure 2D**).

In conclusion, RNA-seq analysis revealed dynamic gene expression changes and time-dependent regulatory patterns in *T. ambiguum* under cold stress, providing a solid foundation for further exploration of the molecular mechanisms and key genes in-volved in cold stress responses.

### 3.4 Dynamic changes in differentially expressed genes under cold stress in *T. ambiguum*

**Figure 3:**
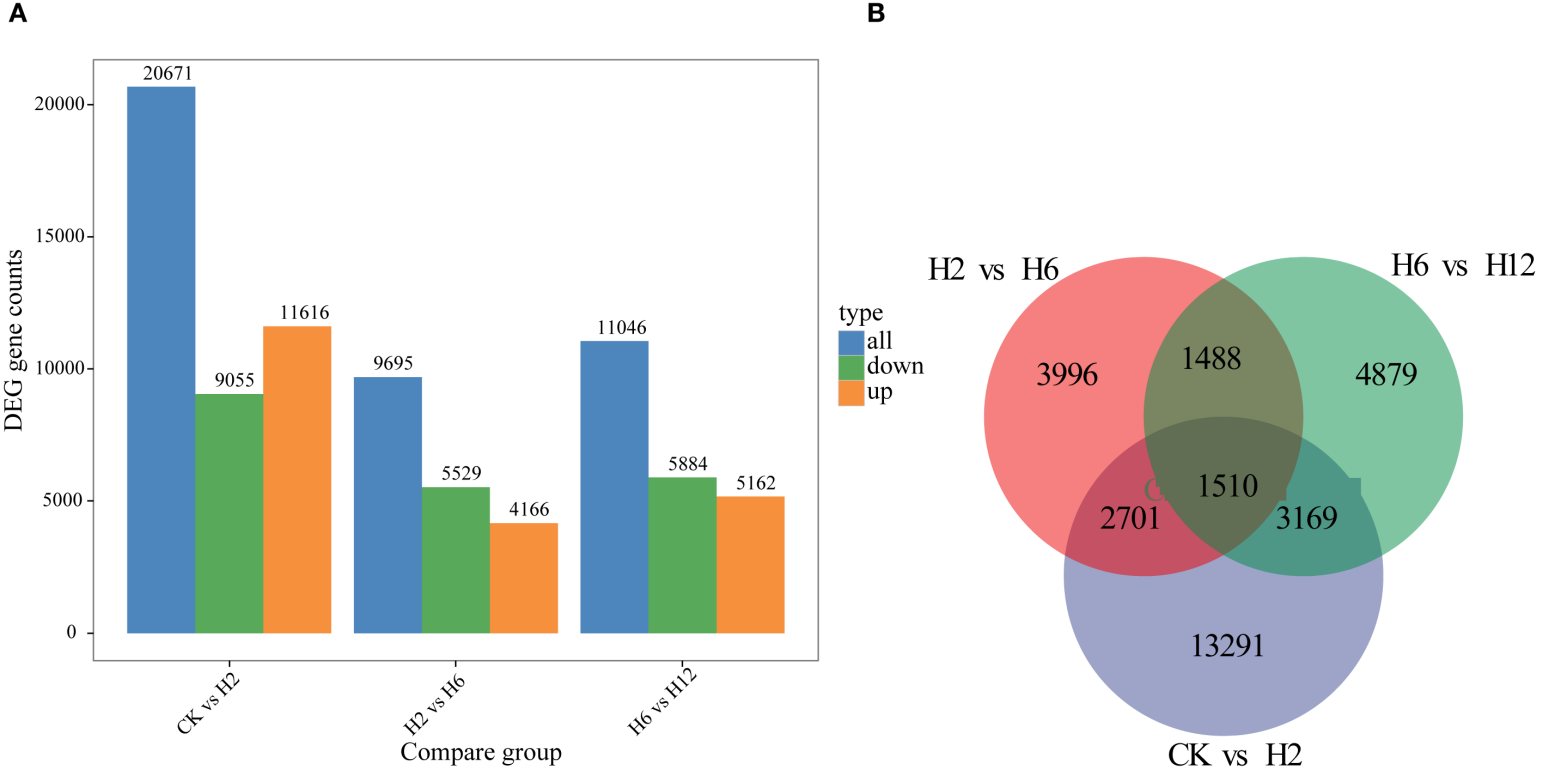
Transcriptomic analysis of differentially expressed genes (DEGs) in *T. ambiguum* under cold stress conditions. **(A)** The number of upregulated and downregulated DEGs across pairwise comparisons of cold stress conditions. **(B)** Venn diagram illustrating the overlap and specificity of DEGs among the three pairwise comparisons.

Cold stress had a profound impact on gene expression patterns, which exhibited dynamic changes as the stress duration increased. In the comparison between CK and H2, the number of differentially expressed genes (DEGs) was the highest, totaling 20,671, with upregulated genes (11,616) predominating. This indicates that in the early stages of cold stress, the plant activated a large number of genes to respond to sudden environmental changes. However, in the comparison between H2 and H6, the number of DEGs decreased to 9,695, with downregulated genes (5,529) exceeding upregulated genes (4,166). This suggests that as the stress persisted, the plant’s response gradually stabilized, and the expression of some genes returned to baseline levels. In contrast, the comparison between H6 and H12 showed an increase in DEGs to 11,046, with upregulated and downregulated genes being relatively balanced (5,162 and 5,884, respectively). This indicates that the plant likely activated new adaptive mechanisms to cope with prolonged cold stress (**Figure 3A**).

Venn diagram analysis revealed further insights into the overlap and specificity of DEGs across treatment groups. The CK vs. H2 comparison exhibited the highest number of specific DEGs (13,291), reflecting the strong disruption of the gene expression profile caused by the initial stress. In contrast, the number of specific genes was lower in the H2 vs. H6 (3,996) and H6 vs. H12 (4,879) comparisons, suggesting that as cold stress persisted, gene expression patterns became increasingly conserved. Moreover, 1,510 DEGs were commonly expressed across all treatment groups, potentially playing central roles in the cold stress response, including processes such as stress perception, signal transduction, and defense mechanisms (**Figure 3B**).

### 3.5 GO functional classification analysis of differentially expressed genes under cold stress in *T. ambiguum*

To further characterize the functional roles of differentially expressed genes (DEGs) in *T. ambiguum* under cold stress, Gene Ontology (GO) enrichment analysis was per-formed on DEGs identified from three pairwise comparisons: CK vs H2, H2 vs H6, and H6 vs H12. The enriched GO terms were categorized into three main functional groups: biological process, cellular component, and molecular function.

**Figure 4:**
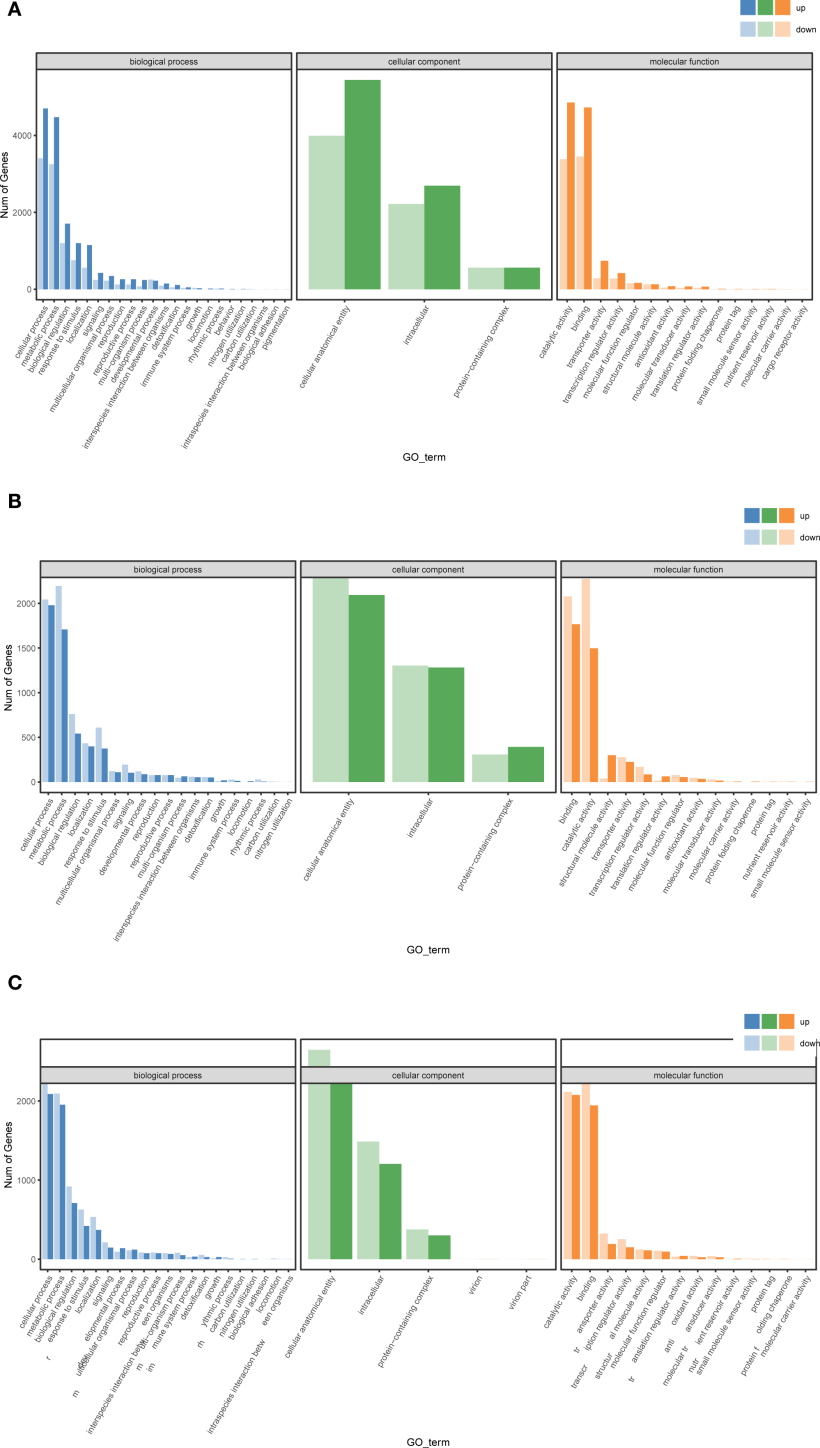
Gene Ontology (GO) enrichment analysis of differentially expressed genes (DEGs) in *T. ambiguum* under cold stress. **(A)** GO enrichment analysis for CK vs H2, **(B)** H2 vs H6, and **(C)** H6 vs H12 comparisons demonstrates distinct functional changes across biological processes, cellular components, and molecular functions, reflecting the dynamic transcriptional responses of *T. am-biguum* to cold stress.

In the CK vs H2 comparison, DEGs in the biological process category were significantly enriched in terms such as “response to stimulus” and “metabolic process,” with the highest number of genes associated with the “oxidation-reduction process.” This suggests that during the early stages of cold stress, plants regulate metabolic and antioxidant responses to mitigate environmental changes. In the cellular component category, many DEGs were associated with “intracellular component” and “mem-brane-related structures,” highlighting the substantial impact of cold stress on cellular integrity. In the molecular function category, DEGs were predominantly enriched in “catalytic activity” and “binding activity,” indicating that cold stress influences cellular physiology by modulating enzymatic functions and molecular interactions (**Figure 4A**).

In the H2 vs H6 comparison, the enriched terms in the biological process category remained focused on “metabolic process” and “response to stimulus,” but a marked increase in terms related to “transport process” was observed. This suggests that as cold stress persisted, the plant enhanced transmembrane transport of substances and energy to maintain homeostasis. In the cellular component category, terms related to the “cytoplasm” were predominant, reflecting active intracellular signaling and metabolic activity. In the molecular function category, “transporter activity” showed significant enrichment, indicating that material transport regulation became critical in response to prolonged cold stress (**Figure 4B**).

In the H6 vs H12 comparison, genes involved in the “secondary metabolic process” were significantly enriched within the biological process category, suggesting that plants activated secondary metabolic pathways to adapt to prolonged cold exposure. In the cellular component category, terms associated with “organelle” were significantly enriched, emphasizing the essential role of organelles in long-term stress adaptation. In the molecular function category, genes related to “oxidoreductase activity” and “ion binding” were notably enriched, likely reflecting regulatory mechanisms involved in maintaining intracellular homeostasis under cold stress (**Figure 4C**).

### 3.6 KEGG pathway enrichment analysis of differentially expressed genes under cold stress in *T. ambiguum*

To further explore the metabolic pathways and molecular regulatory mechanisms of *T. ambiguum* under cold stress at different stages, KEGG pathway enrichment analysis was performed on the differentially expressed genes (DEGs) identified in three pairwise comparisons: CK vs H2, H2 vs H6, and H6 vs H12. The analysis revealed that cold stress significantly affected multiple biological processes, with pathway enrichment patterns dynamically changing as stress duration increased.

**Figure 5:**
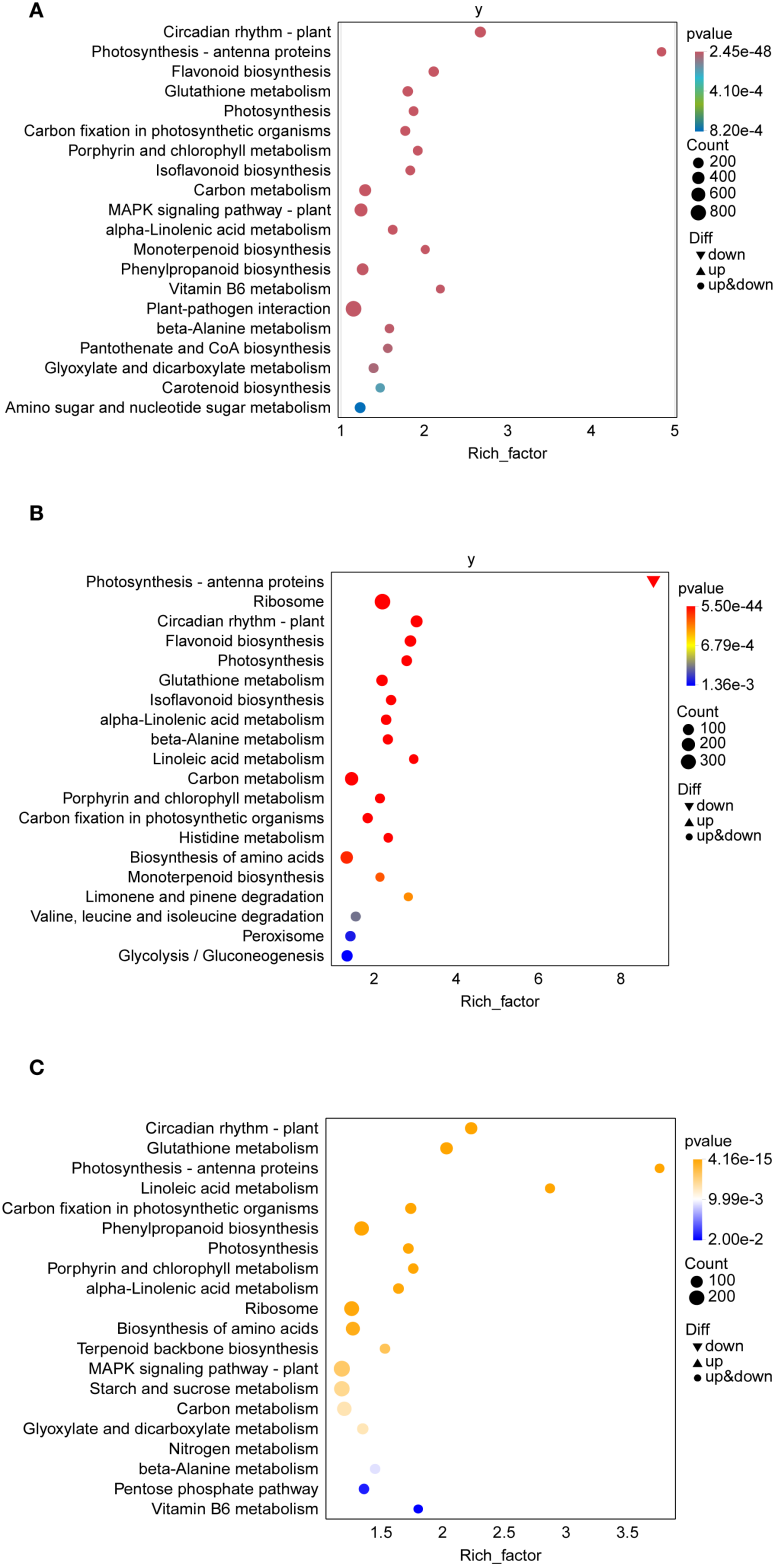
KEGG pathway enrichment analysis of differentially expressed genes (DEGs) in T. am-biguum under cold stress conditions. **(A)** KEGG pathway enrichment for CK vs H2. **(B)** KEGG pathway enrichment for H2 vs H6. **(C)** KEGG pathway enrichment for H6 vs H12. Bubble sizes represent the number of genes enriched in each pathway, while colors indicate the significance (*p*-value) of enrichment. Pathways include those associated with metabolic, signaling, and stress-responsive processes.

In the CK vs H2 comparison, the significantly enriched pathways included “photosynthesis,” “photosynthesis antenna proteins,” and “carbon fixation in photosynthetic organisms.” These results indicate that, in the early stage of cold stress, plants prioritized the regulation of photosynthesis-related metabolic pathways to maintain photo-synthetic efficiency and carbon metabolism balance under sudden environmental changes. Additionally, the enrichment of antioxidant-related pathways, such as “glutathione metabolism,” and secondary metabolism-related pathways, such as “flavonoid biosynthesis,” suggested that plants rapidly activated antioxidant defense mechanisms to counteract reactive oxygen species (ROS) accumulation. The enrichment of the “MAPK signaling pathway” highlighted the importance of signal perception and transduction in the early cold stress response (**Figure 5A**).

In the H2 vs H6 comparison, pathway enrichment shifted toward the regulation of basic metabolism and signal transduction. Key enriched pathways included “carbon metabolism” and “biosynthesis of amino acids,” indicating that prolonged cold stress reprogrammed metabolic activities to maintain cellular homeostasis. Additionally, the significant enrichment of “linoleic acid metabolism” and “beta-alanine metabolism” suggested that plants modulated lipid and amino acid metabolism to stabilize cell membranes and ensure energy supply. Compared to the early stage, the significance of antioxidant and signal transduction pathways decreased, implying that plants gradually adapted to the cold stress environment during this phase (**Figure 5B**).

In the H6 vs H12 comparison, enriched pathways reflected features of long-term stress adaptation. Secondary metabolism-related pathways, such as “phenylpropanoid biosynthesis” and “terpenoid backbone biosynthesis,” were significantly enriched, suggesting that plants activated secondary metabolic pathways to enhance stress tolerance. Additionally, the enrichment of pathways related to “ribosome” and “nitrogen metabolism” indicated that plants adjusted protein synthesis and nitrogen metabolism to adapt to prolonged cold stress. These results suggest that plants developed more complex regulatory patterns to maintain metabolic balance under sustained stress conditions (**Figure 5C**).

The KEGG pathway enrichment analysis across different stages revealed the dynamic regulatory features of *T. ambiguum* under cold stress, transitioning from an initial rapid response to long-term adaptation. In the early stage, the focus was on photosynthesis, antioxidant activity, and signal transduction. During the mid-stage, metabolic activities were reprogrammed to stabilize cellular functions. In the later stage, secondary metabolism and protein metabolism were activated to enhance long-term adaptation to cold stress. These findings provide valuable insights into the molecular mechanisms underlying cold stress responses and lay a foundation for future research.

### 3.7 Trend analysis of differentially expressed genes in *T. ambiguum* under cold stress

This study applied trend analysis to categorize differentially expressed genes (DEGs) in *T. ambiguum* under cold stress across three stages: early (CK vs H2), mid (H2 vs H6), and late (H6 vs H12). By analyzing the trends in upregulated and downregulated gene expression, we identified the plant’s dynamic regulatory strategies, including rapid response, metabolic resource optimization, and energy conservation, providing key insights into the molecular mechanisms of cold stress adaptation.

In the early stage of cold stress (CK vs H2), DEGs in *T. ambiguum* exhibited substantial changes, with genes categorized into two trend modules. Module 0 displayed a pronounced upregulation trend, suggesting that the plant rapidly reprograms gene expression to respond to sudden environmental stress. KEGG enrichment analysis revealed significant enrichment in antioxidant metabolism-related pathways, including “glutathione metabolism” and “ascorbic acid and galacturonic acid metabolism,” indicating enhanced antioxidant capacity to counteract oxidative stress induced by cold conditions. Additionally, pathways such as “cysteine and methionine metabolism” and “thiamine metabolism” were significantly enriched, suggesting that plants regulate key metabolite synthesis and utilization to maintain cellular homeostasis and strengthen stress resistance. Enrichment of “photosynthesis” and related pathways (e.g., “photosynthesis antenna proteins”) suggests that, despite the inhibitory effects of low temperatures, the plant adjusts photosynthesis related genes to optimize energy metabolism, thereby supporting growth and survival. Furthermore, enrichment of the “phosphatidylinositol signaling system” indicates that plants rapidly perceive environmental changes and activate adaptive responses via signal transduction during the early cold stress stage.

In the mid-stage of cold stress (H2 vs H6), Module 0 exhibited a significant downregulation trend, reflecting the plant’s strategy to suppress the expression of specific genes, reduce metabolic activity, conserve energy, and optimize resource allocation. KEGG enrichment analysis revealed significant enrichment in pathways such as “photosynthesis,” “photosynthesis antenna proteins,” and “carbon fixation,” suggesting that the plant suppresses photosynthesis-related genes to minimize energy consumption while maintaining carbon fixation to support basic metabolic functions. Enrichment in pathways such as “pentose phosphate pathway,” “glycolysis/gluconeogenesis,” and “carbon metabolism” indicates that the plant optimizes carbon utilization by dynamically regulating carbon metabolism-related genes, ensuring physiological functions under stress. Antioxidant-related pathways, including “glutathione metabolism” and “ascorbic acid and galacturonic acid metabolism,” were also enriched, suggesting that the plant enhances antioxidant capacity to mitigate oxidative damage. Additionally, enrichment in secondary metabolism pathways such as “flavonoid biosynthesis” and “sesquiterpene and triterpene synthesis” indicates that plants accumulate secondary metabolites to strengthen cold tolerance. Pathways related to “amino acid biosynthesis” and “alanine, aspartate, and glutamate metabolism” were also enriched, indicating that plants regulate amino acid metabolism to balance protein synthesis and provide supplementary energy for stress adaptation.

**Figure 6:**
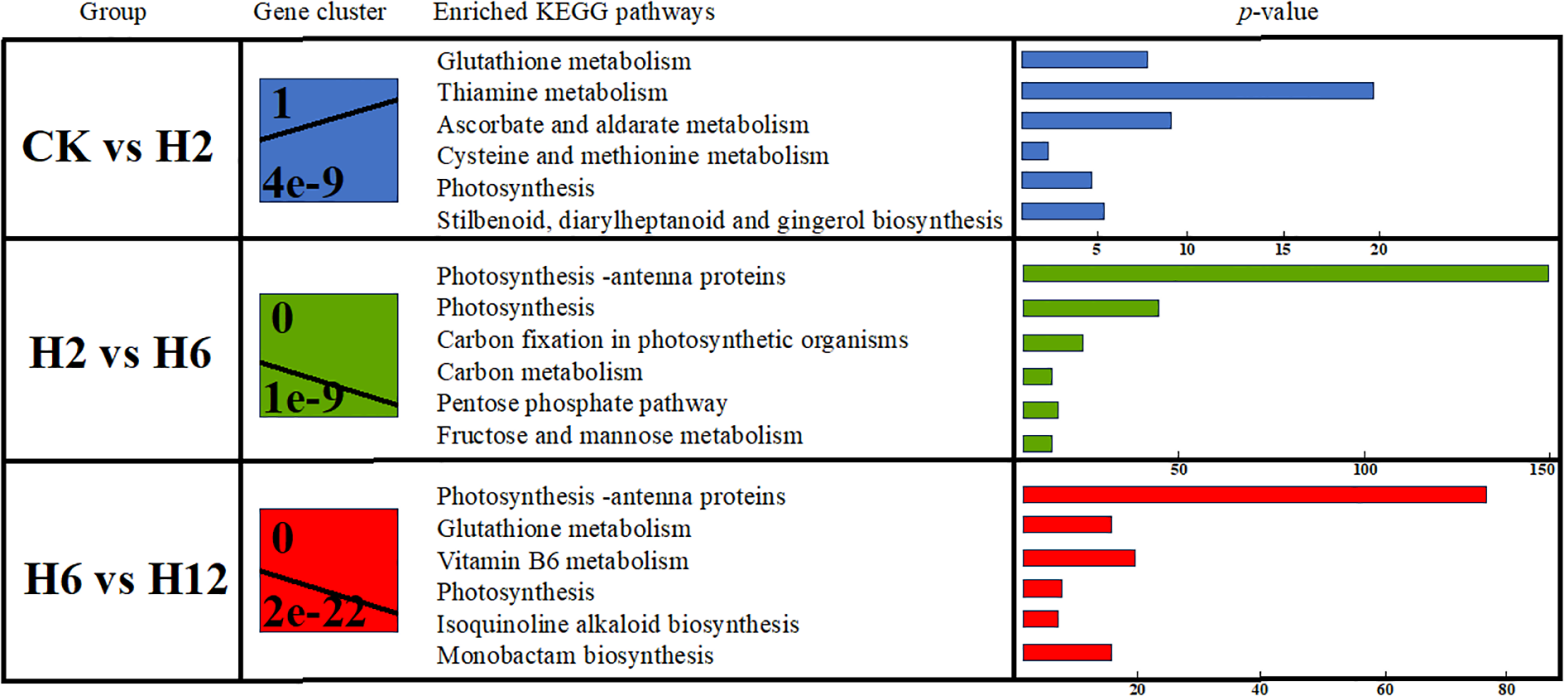
Trend analysis of KEGG pathway enrichment in *T. ambiguum* under cold stress. This figure presents the trend analysis of KEGG pathway enrichment for different comparison groups (CK vs H2, H2 vs H6, H6 vs H12). “Gene cluster” represents the significant expression trend modules identified for each group. “Enriched KEGG pathways” lists the pathways enriched within these modules, and the corresponding *p*-values indicate the statistical significance of each enriched pathway. Bar lengths represent the number of genes involved in each pathway.

In the late stage of cold stress (H6 vs H12), Module 0 exhibited a marked downregulation trend, reflecting the plant’s strategy to suppress specific metabolic genes, reduce metabolic activity, conserve energy, and optimize resource allocation under prolonged cold stress. Antioxidant-related pathways, including “glutathione metabolism” and “vitamin B6 metabolism,” were significantly enriched, indicating that the plant maintains cellular homeostasis and mitigates oxidative damage through sustained antioxidant activity, despite overall metabolic downregulation. Enrichment in pathways related to “sulfur metabolism” and “nitrogen metabolism” suggests that the plant adjusts sulfur and nitrogen metabolic pathways to enhance environmental adaptability. Furthermore, enrichment in signal transduction pathways, such as “phosphatidylinositol signaling system” and “SNARE-mediated vesicle transport,” alongside pathways related to gene expression regulation, including “mRNA surveillance” and “aminoacyl-tRNA biosynthesis,” suggests that, despite overall metabolic downregulation, the plant sustains precise regulatory mechanisms for signal transduction and gene translation to adapt to cold stress (**Figure 6**).

In summary, *T. ambiguum* exhibits dynamic regulation of gene expression across different stages of cold stress, transitioning from rapid responses in the early stage to metabolic resource optimization in the mid-stage and reduced metabolic activity in the late stage, highlighting a multi-layered adaptive strategy. The plant coordinates key pathways such as photosynthesis, carbon metabolism, antioxidant metabolism, secondary metabolism, and signal transduction to provide a strong molecular foundation for coping with cold stress, while also offering valuable insights into the molecular regulatory networks underlying cold stress responses.

### 3.8 Validation by quantitative real-time PCR

**Figure 7:**
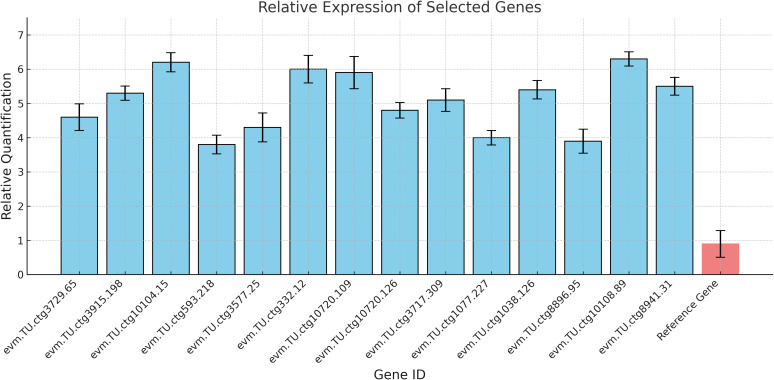
qRT-PCR verification of differentially expressed genes.

To verify the consistency of the RNA-seq data, 15 randomly selected differentially expressed genes (DEGs) were validated using qRT-PCR. As shown in the figure, the qRT-PCR results were consistent with the RNA-seq expression data, confirming the accuracy of the transcriptome sequencing results (**Figure 7**).

## 4 Discussion

### 4.1 Physiological adaptations of *T. ambiguum* to cold stress

Physiological indicators serve as direct reflections of plant responses to environmental stress and can complement transcriptomic data to provide a more comprehensive understanding of adaptive mechanisms. In the present study, *T. ambiguum* showed distinct physiological changes under cold stress, highlighting the roles of osmotic regulation and antioxidative defense in its tolerance strategy (Amin et al., 2021). The early and significant increase in soluble protein (SP) content at 2 h suggests that *T. ambiguum* rapidly enhances its protein synthesis or stabilizes existing proteins to cope with stress-induced damage. This is consistent with the transcriptomic enrichment of genes related to protein folding and processing, such as heat shock proteins (HSPs) and chaperones, which help maintain protein homeostasis under stress (Ding et al., 2024).

Malondialdehyde (MDA), a marker of lipid peroxidation, peaked at 6 h, indicating that prolonged cold exposure leads to oxidative damage to cell membranes. This correlates with the upregulation of genes involved in lipid metabolism and membrane stabilization observed in the RNA-seq data, suggesting that *T. ambiguum* responds to oxidative stress not only by scavenging reactive oxygen species (ROS) but also by repairing or reinforcing membrane integrity (Gao et al., 2024a). The continuous accumulation of soluble sugars (SS) and proline (Pro) throughout the treatment indicates their dual roles in osmotic adjustment and ROS scavenging. These small molecules are known to stabilize proteins and membranes, and their sustained elevation suggests that *T. ambiguum* maintains cellular turgor and minimizes dehydration damage under low temperatures (Gu et al., 2021). Their roles are further supported by the enrichment of carbohydrate metabolism and proline biosynthesis pathways in the transcriptome (Wang et al., 2022).

Antioxidant enzymes also played a pivotal role in the early defense response. The marked increases in catalase (CAT), peroxidase (POD), and superoxide dismutase (SOD) activities at 2 h demonstrate a rapid activation of ROS detoxification mechanisms (Hussain et al., 2023). These physiological changes are in line with transcriptomic data showing upregulation of genes encoding antioxidant enzymes, including CAT, POD, and SOD gene families, as well as related transcription factors such as WRKY and NAC. Interestingly, CAT activity decreased significantly in the later stages, possibly due to enzyme inactivation or feedback regulation under sustained stress (Li et al., 2024). In contrast, POD and SOD activities remained relatively high at 6 h, suggesting a shift in the dominant antioxidative mechanism as stress progressed (Mazur et al., 2024). These temporal patterns reflect a finely tuned antioxidant system that adjusts its components depending on the stage and intensity of cold stress (Niu et al., 2020).

Overall, the dynamic physiological responses of *T. ambiguum* under cold stress—particularly in osmolyte accumulation and antioxidant enzyme activity—strongly support the transcriptomic findings and underscore the coordinated regulation between molecular and biochemical pathways. These results provide physiological validation for the candidate genes and regulatory networks identified, and collectively contribute to understanding the cold tolerance strategy of this legume species.

### 4.2 Dynamic molecular response mechanisms of *T. ambiguum* under cold stress

This study utilized high-quality transcriptomic data and a multi-timepoint dynamic experimental design to comprehensively analyze the molecular responses of *T. ambiguum* under cold stress, offering novel insights into its cold tolerance mechanisms. A core strength of this study lies in the high-quality data, with Q30 sequencing values exceeding 97% and stable GC content ranging between 41.25% and 41.75%, which aligns well with the genomic characteristics of *T. ambiguum*. Furthermore, the high correlation between biological replicates (PCC > 0.98) underscores the reliability of the experimental design. The data quality is comparable to studies on plants such as *Cucumis sativus* (Sun et al., 2024) and *Chrysanthemum morifolium* (Wu et al., 2024), and surpasses transcriptomic studies with low replication or data bias, providing a strong foundation for elucidating dynamic gene expression changes. Additionally, the multi-timepoint sampling design (2 hours, 6 hours, and 12 hours) represents a distinct advantage over single-timepoint analyses, allowing for a detailed exploration of temporal and phase-specific gene ex-pression dynamics.

This study uncovered significant temporal variations in the number and functional categories of differentially expressed genes (DEGs) across timepoints, highlighting dynamic changes in gene expression. The highest number of DEGs (20,671) was observed at the early stage (H2), suggesting that the plant rapidly activates transcriptional reprogramming to mitigate cell damage and metabolic stress induced by cold stress (Chinnusamy et al., 2007). This rapid response mechanism is consistent with findings in *Nicotiana tabacum* (Jin et al., 2017) and *Actinidia deliciosa* (Sun et al., 2021), where early-stage cold stress led to upregulation of genes related to antioxidant enzymes and membrane stability. At the mid-stage (H6), the number of DEGs decreased (9,695), indicating that the plant was gradually adapting to the stress environment, resulting in the stabilization of gene expression. This adaptive stage is consistent with findings from studies on mid-stage stress responses in *Xanthoceras sorbifolia* (Wang et al., 2020) and *Medicago sativa* (Liu et al., 2024), suggesting conserved mechanisms across species. At the late stage (H12), the number of DEGs increased again (11,046), indicating that the plant activated novel adaptive mechanisms under prolonged cold stress, such as the upregulation of secondary metabolism-related genes to strengthen antioxidant defense and structural protection.

Gene Ontology (GO) functional enrichment analysis further revealed the stage-specific response mechanisms of the DEGs. At the early stage, cold stress significantly induced GO terms related to antioxidant defense and signal perception, such as “oxidation-reduction process” and “response to stimulus.” These findings align with studies on *Chrysanthemum morifolium* (Wu et al., 2024) and *Arachis hypogaea* (Wang et al., 2021), where antioxidant pathways were prominently activated under cold stress. During the mid-stage, significant enrichment in GO terms such as “transport process” and “transmembrane transporter activity” highlighted the importance of transmembrane transport in material and energy regulation, particularly for lipid metabolism and membrane stability, as observed in *Medicago sativa* (Liu et al., 2024) and *Brassica napus* (Zhu et al., 2024). At the late stage, the significant upregulation of secondary metabolism-related genes, particularly those involved in “flavonoid biosynthesis” and “phenylpropanoid metabolism,” suggested that the plant enhanced its stress resistance and cellular protection through metabolic remodeling. This stage-specific pattern is consistent with findings in *Actinidia deliciosa* (Sun et al., 2021) and *Cucurbita maxima* (Li et al., 2021), underscoring the central role of secondary metabolism in long-term cold adaptation.

KEGG pathway analysis revealed a dynamic shift in metabolic pathways from early to late stages of cold stress. At the early stage, pathways related to photosynthesis and carbon fixation were significantly enriched, indicating that the plant prioritizes energy metabolism to mitigate the initial effects of stress. Similar patterns have been observed in *Oryza sativa* (Fu et al., 2023) and *Chenopodium quinoa* (Xie et al., 2022), where optimization of carbon metabolism was crucial for alleviating short-term stress. As the stress progressed, secondary metabolic pathways, such as flavonoid and phenylpropanoid metabolism, became predominant during the mid- to late stages, signaling a transition from energy metabolism to cellular protection mechanisms for long-term adaptation (Waadt et al., 2022). Hormone signaling pathways, particularly abscisic acid (ABA) and jasmonic acid (JA) signaling, were also significantly enriched, highlighting their regulatory roles in cold stress. ABA signaling enhances cellular protection by inducing antioxidant enzyme gene expression and reducing water loss through stomatal regulation, while JA signaling promotes secondary metabolism-related gene expression, bolstering chemical defenses. These mechanisms have been confirmed in studies on *Chrysanthemum morifolium* and *Nicotiana tabacum* (Eremina et al., 2016).

Compared to other leguminous plants, such as *Medicago sativa* (Liu et al., 2024) and *Phaseolus vulgaris* (Yang et al., 2023), *T. ambiguum* exhibited more pronounced differential gene expression and metabolic pathway regulation under cold stress, reflecting its greater sensitivity and adaptability to low temperatures. This superior cold tolerance is likely associated with its deep root system and unique ecological adaptations, offering strong support for its potential use in cold-resilient agricultural systems.

### 4.3 Molecular mechanisms of trend-based regulatory analysis in *T. ambiguum* under cold stress

This study employs trend analysis to explore the dynamic gene expression regulation in *T. ambiguum* under cold stress. The classification of gene expression trends at the early (CK vs H2), mid (H2 vs H6), and late (H6 vs H12) stages reveals the multi-layered regulatory strategy of plants, shifting from rapid response to long-term adaptation. These findings not only elucidate the stage-specific metabolic dynamics underlying the plant’s response to cold stress but also offer valuable comparisons and insights into the molecular regulatory mechanisms of other cold-resistant plants.

In the early stage (CK vs H2), differentially expressed genes (DEGs) in *T. ambiguum* show a significant upregulation, suggesting that the plant quickly reprograms gene expression to adapt to sudden cold stress. KEGG pathway enrichment analysis at this stage demonstrates significant activation of antioxidant-related pathways, such as “glutathione metabolism” and “ascorbic acid and galacturonic acid metabolism,” highlighting the plant’s rapid scavenging of reactive oxygen species (ROS) to prevent oxidative damage (Gusain et al., 2023). This rapid antioxidant response aligns closely with mechanisms observed in *Nicotiana tabacum* (Jin et al., 2017) and *Actinidia deliciosa* (Sun et al., 2021). Furthermore, genes involved in cysteine, methionine, and thiamine metabolism are significantly upregulated, indicating that the plant enhances the synthesis and utilization of metabolites to maintain cellular homeostasis and increase cold tolerance. At this stage, the plant prioritizes the upregulation of photosynthesis-related genes (e.g., “photosynthetic antenna proteins”), suggesting that despite the potential inhibition of photosynthetic efficiency by cold stress, the plant regulates these genes to maintain basal energy metabolism (Ding and Yang, 2022). Similar photosynthesis optimization strategies have been reported in *Oryza sativa* (Fu et al., 2023) and *Chenopodium quinoa* (Xie et al., 2022). Furthermore, the activation of signaling pathways, such as the “phosphatidylinositol signaling system,” indicates that plants rapidly perceive environmental changes and initiate adaptive responses, aligning with cold stress response studies in *Triticum aestivum* (Lv et al., 2022) and *Cocos nucifera* (Lu et al., 2023).

During the mid-phase (H2 vs H6), the overall expression trend of DEGs shifted from significant upregulation to partial downregulation, indicating a transition from the rapid response phase to metabolic resource optimization and energy conservation. The downregulation of photosynthesis-related genes (such as “photosynthetic antenna proteins” and “carbon fixation”) indicates that the plant actively suppresses energy-intensive metabolic processes to conserve energy while maintaining a fundamental balance in carbon metabolism (Yow et al., 2023). This strategy of metabolic resource optimization has also been observed in the mid-phase stress responses of *Vicia sativa* (Rui et al., 2022) and *Anthurium andraeanum* (Dou et al., 2023). Moreover, the sustained activation of antioxidant-related pathways (such as “glutathione metabolism” and “ascorbate metabolism”) suggests that, despite the overall decline in metabolic activity, the plant prioritizes antioxidant defense to mitigate cellular damage induced by stress. Additionally, the significant enrichment of secondary metabolism-related pathways (such as “flavonoid biosynthesis” and “sesquiterpene and triterpene biosynthesis”) indicates that the plant enhances its cold tolerance by accumulating antioxidant secondary metabolites (Wang et al., 2024). This mechanism aligns with findings from secondary metabolism studies in *Zea mays* (Waititu et al., 2021) and *Eremochloa ophiuroides* (Liu et al., 2023), further corroborating the essential role of secondary metabolism in cold adaptation.

During the later phase (H6 vs H12), the expression of DEGs showed a significant downregulation, reflecting the plant’s strategy to conserve energy by decreasing overall metabolic activity and reallocating resources in response to sustained cold stress. The continuous downregulation of photosynthesis-related pathways (such as “photosynthetic antenna proteins”) indicates the plant’s metabolic suppression strategy under prolonged stress, aiming to reduce unnecessary energy consumption (Aslam et al., 2022). Additionally, despite the overall decline in metabolic activity, the significant enrichment of antioxidant-related pathways (such as “glutathione metabolism” and “vitamin B6 metabolism”) suggests that the plant maintains its antioxidant defense mechanisms to ensure cellular homeostasis (Kidokoro et al., 2022). The maintenance of long-term antioxidant metabolism has been similarly validated in studies on *Vigna radiata* (Chen et al., 2008) and *Glycine max* (Muncan et al., 2022). Concurrently, the activation of signaling and molecular transport pathways (such as the “phosphoinositide signaling system” and “SNARE-mediated vesicle transport”), alongside pathways associated with gene expression regulation (such as the “mRNA surveillance pathway” and “aminoacyl-tRNA biosynthesis”), indicates that the plant precisely regulates gene translation and signaling even amid an overall decline in metabolic activity to adapt to the cold stress environment (Manasa et al., 2022). This finding underscores the critical role of cellular-level regulation in long-term stress adaptation.

During the early phase of cold stress, *T. ambiguum* undergoes rapid gene expression reprogramming, adapts in the intermediate phase by optimizing metabolic resources and accumulating secondary metabolites, and in the later phase, it conserves energy and strengthens long-term adaptation by reducing metabolic activity and sustaining antioxidant defense. These phase-specific regulatory mechanisms reveal the complex network underlying the plant’s transition from short-term defense to long-term adaptation and offer critical insights for further unraveling the molecular mechanisms of cold stress.

## 5 Conclusions

This study provides a comprehensive evaluation of the physiological and molecular responses of *T. ambiguum* under cold stress, highlighting the plant’s adaptive mechanisms. The physiological analysis revealed dynamic changes in soluble protein (SP), proline (Pro), soluble sugars (SS), and antioxidant enzyme activities (CAT, POD, and SOD) over time. These changes indicate the plant’s ability to rapidly scavenge reactive oxygen species (ROS), maintain osmotic balance, and protect its cellular membranes, demonstrating its robust biochemical defense mechanisms.

To further understand the molecular basis of these responses, high-throughput RNA sequencing (RNA-seq) was performed at multiple time points during cold stress. The transcriptomic analysis revealed a temporally phased response to cold stress in *T. ambiguum*. In the early stage, genes related to antioxidant defense were upregulated to mitigate oxidative damage, while energy-generating processes, such as photosynthesis, were maintained to support cellular function. In the mid-stage, a suppression of energy-intensive processes was observed, suggesting a reallocation of resources to sustain stress tolerance. In the late stage, pathways associated with flavonoid and phenylpropanoid biosynthesis were significantly enriched, contributing to cellular protection and adaptation to prolonged cold stress.

These findings, supported by both physiological and transcriptomic data, offer valuable insights into the multi-layered adaptive strategies of *T. ambiguum* under cold stress. By integrating the physiological and molecular responses, this study not only deepens our understanding of cold stress adaptation in *T. ambiguum* but also provides a foundation for improving cold tolerance in leguminous forage crops through molecular breeding. The results underline the importance of key molecular pathways, including antioxidant defense, osmotic regulation, and secondary metabolism, in facilitating cold stress adaptation and offer a basis for future breeding programs aimed at enhancing cold tolerance in forage crops.

## Data Availability

The datasets presented in this study can be found in online repositories. The names of the repository/repositories and accession number(s) can be found below: https://www.ncbi.nlm.nih.gov/, PRJNA1232370.

## Supplementary material

Supplementary Table 1 Quality assessment of transcriptome sequencing data for *Trifolium ambiguum* under cold stress.

Supplementary Table 2 qRT-PCR primer information.

Supplementary Table 3 Quality Assessment of Transcriptome Sequencing Data for *T. ambiguum* under Cold Stress.
