# Correction: Cold stress adaptation in *Trifolium ambiguum*: physiological and transcriptomic insights

**DOI:** 10.3389/fpls.2025.1731542

**Published:** 2025-12-04

**Authors:** Kefan Cao, Huimin Zhang, Yiming Ma, Fan Huang

**Affiliations:** 1 Institute of Grassland Research, Chinese Academy of Agricultural Science, Hohhot, China; 2 College of Grassland Science/Key Laboratory of Grassland Resources of Ministry of Education, Inner Mongolia Agricultural University, Hohhot, China

**Keywords:** *Trifolium ambiguum* M. Bieb, cold stress, antioxidant enzymes, osmotic adjustment, RNA-seq

In the previously published article, there was a mistake in the order of authors’ affiliation. The affiliations in the published original version of the article were interchanged. The affiliations in the published original version of the article were: “^1^College of Grassland Science/Key Laboratory of Grassland Resources of Ministry of Education, Inner Mongolia Agricultural University, Hohhot, China”, “^2^Institute of Grassland Research, Chinese Academy of Agricultural Science, Hohhot, China”. The correct order should be: “^1^Institute of Grassland Research, Chinese Academy of Agricultural Science, Hohhot, China”, “^2^College of Grassland Science/Key Laboratory of Grassland Resources of Ministry of Education, Inner Mongolia Agricultural University, Hohhot, China”. The affiliations assigned in the published original version of the article were: “Kefan Cao^1,2^, Huimin Zhang^1^, Yiming Ma^1^, Fan Huang^2*”^. The correct affiliations assigned should be: “Kefan Cao^1,2^, Huimin Zhang^2^, Yiming Ma^2^, Fan Huang^1*”^.

The original article has been updated.

