# Supplementary figures and images for: Cold stress adaptation in Trifolium ambiguum: physiological and transcriptomic insights
Source: Front Plant Sci. 2025 Oct 16;16:1645123. doi: 10.3389/fpls.2025.1645123 (PMC12571821; doi:10.3389/fpls.2025.1645123)

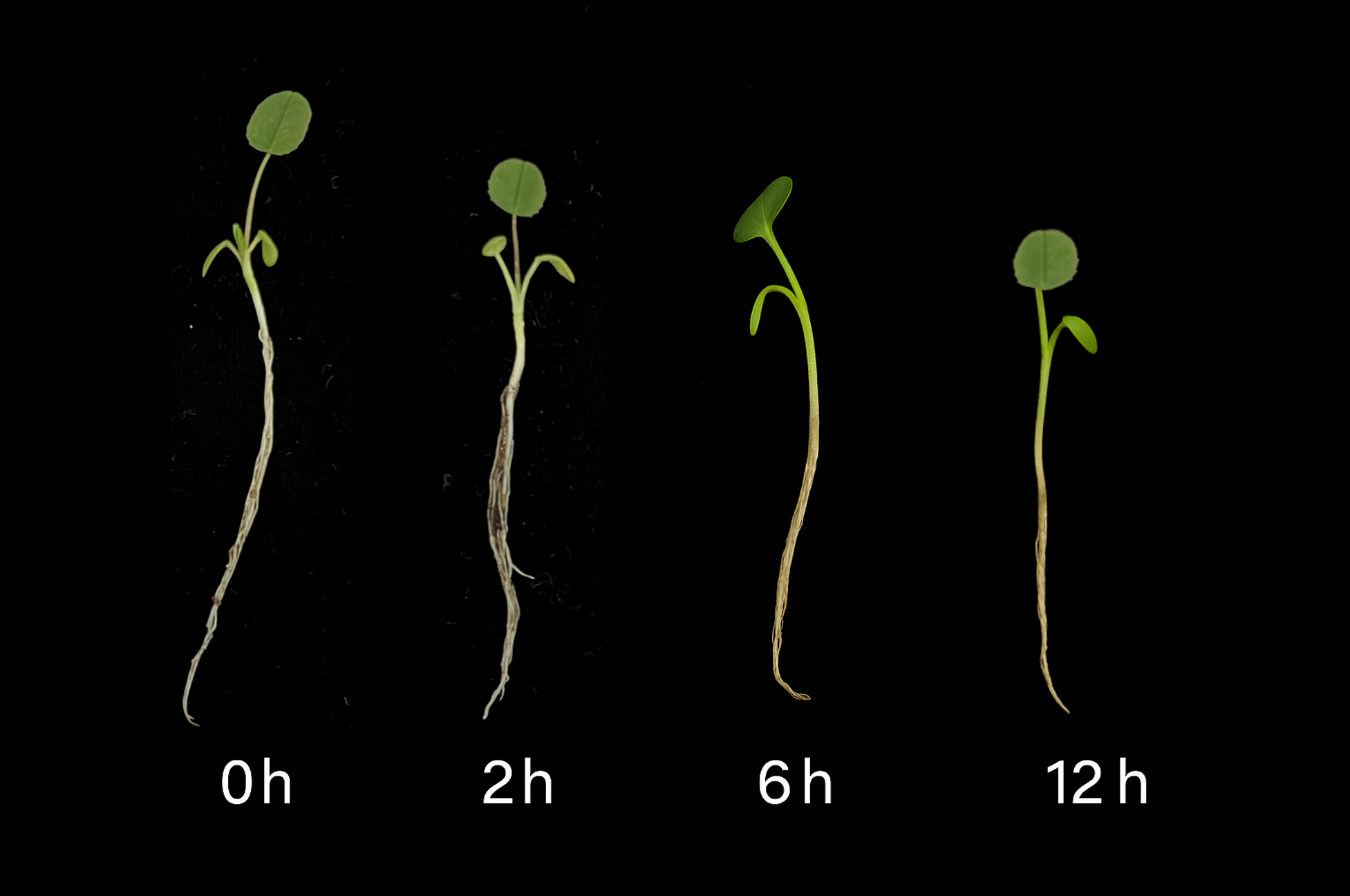

Supplement: Supplementary Table 1 — Quality assessment of transcriptome sequencing data for Trifolium ambiguum under cold stress. [file Image1.png]
