# Supplementary material for: Cold stress adaptation in Trifolium ambiguum: physiological and transcriptomic insights
Source: Front Plant Sci. 2025 Oct 16;16:1645123. doi: 10.3389/fpls.2025.1645123 (PMC12571821; doi:10.3389/fpls.2025.1645123)
Supplement: Supplementary Table 2 — qRT-PCR primer information. [file Table1.docx]

Supplementary Material

**Supplementary Table 1.** Sample-to-sample Pearson correlation coefficients among RNA-seq datasets of *Trifolium ambiguum* seedlings under cold stress (0 h, 2 h, 6 h, and 12 h).

|  | CK1 | CK2 | CK3 | H2-2 | H2-1 | H2-3 | H6-3 | H6-1 | H6-2 | H12-2 | H12-1 | H12-3 |
| --- | --- | --- | --- | --- | --- | --- | --- | --- | --- | --- | --- | --- |
| CK1 | 1 | 0.971 | 0.965 | 0.528 | 0.508 | 0.512 | 0.246 | 0.25 | 0.252 | 0.254 | 0.272 | 0.25 |
| CK2 | 0.971 | 1 | 0.972 | 0.518 | 0.506 | 0.507 | 0.242 | 0.241 | 0.248 | 0.245 | 0.263 | 0.245 |
| CK3 | 0.965 | 0.972 | 1 | 0.519 | 0.511 | 0.51 | 0.24 | 0.242 | 0.248 | 0.243 | 0.263 | 0.245 |
| H2-2 | 0.528 | 0.518 | 0.519 | 1 | 0.954 | 0.944 | 0.782 | 0.803 | 0.8 | 0.688 | 0.733 | 0.695 |
| H2-1 | 0.508 | 0.506 | 0.511 | 0.954 | 1 | 0.956 | 0.795 | 0.803 | 0.81 | 0.723 | 0.733 | 0.717 |
| H2-3 | 0.512 | 0.507 | 0.51 | 0.944 | 0.956 | 1 | 0.8 | 0.791 | 0.8 | 0.723 | 0.733 | 0.717 |
| H6-3 | 0.246 | 0.242 | 0.24 | 0.782 | 0.795 | 0.8 | 1 | 0.936 | 0.95 | 0.828 | 0.824 | 0.817 |
| H6-1 | 0.25 | 0.241 | 0.242 | 0.803 | 0.803 | 0.791 | 0.936 | 1 | 0.954 | 0.815 | 0.835 | 0.833 |
| H6-2 | 0.252 | 0.248 | 0.248 | 0.792 | 0.81 | 0.8 | 0.95 | 0.954 | 1 | 0.828 | 0.859 | 0.846 |
| H12-2 | 0.254 | 0.245 | 0.243 | 0.688 | 0.723 | 0.723 | 0.828 | 0.815 | 0.828 | 1 | 0.942 | 0.929 |
| H12-1 | 0.272 | 0.263 | 0.263 | 0.733 | 0.733 | 0.733 | 0.824 | 0.835 | 0.859 | 0.942 | 1 | 0.958 |
| H12-3 | 0.25 | 0.245 | 0.245 | 0.695 | 0.717 | 0.717 | 0.817 | 0.833 | 0.846 | 0.929 | 0.958 | 1 |

**Supplementary Table 2.** qRT-PCR primer information.

| **NO.** | **Gene ID** | **Forward Primer** | **Reverse Primer** |
| --- | --- | --- | --- |
| 1 | *evm.TU.ctg3729.65* | ATGGCTGCTTCTTCCAACAC | AGCTCATGAGACGTTCAATG |
| 2 | *evm.TU.ctg8859.158* | CAAAGACAGTTTGTGTCACG | TCTTGGATGGTGGCATGGAC |
| 3 | *evm.TU.ctg10104.25* | CTTCCTCAACTCCCTCACCT | GAAGTTGATTCGGCGTCGAT |
| 4 | *evm.TU.ctg5993.28* | AGAAGACAAGCTTTGGAGAG | CCCAATAGGGTACACTTTCT |
| 5 | *evm.TU.ctg3507.25* | TTTGGCCTCTGGTTTGGTTC | ACTGGTTCGGTGGCTACAAC |
| 6 | *evm.TU.ctg6312.12* | ACCCTCCAATTTCCAAAGTC | CTATGGTACCAATTACGAGG |
| 7 | *evm.TU.ctg4592.109* | ATGGGGGGTCTTTGTTCTAA | CCCTCCTTTACACTTGTCAA |
| 8 | *evm.TU.ctg7070.226* | CTTCACTTCACTCACTCACT | AGCGAGATGCTTTCAATGAG |
| 9 | *evm.TU.ctg4437.300* | ATGGGCACTGTGATTGACTC | CAAATTTGAGGAGTGCAGTG |
| 10 | *evm.TU.ctg5993.15* | CTGTTTTCAGGAGAGTTACC | GAACTCCCCGAAGTGTGAAA |
| 11 | *evm.TU.ctg1707.227* | CCATCTAACCAAACCCGACG | AACTCAACTTCGTCGTCAGG |
| 12 | *evm.TU.ctg11098.136* | GAGTTGTGCACTTAGATTGC | CGCTAGATGAGAATAGGAGA |
| 13 | *evm.TU.ctg3686.26* | TTGGTGGAGCTTTTTGTGAG | CTCCCTTTGCCAAATCCAAA |
| 14 | *evm.TU.ctg10149.89* | ATGGCGAGTAAAAGTGCTGA | TTTCCCGAGCTAGCATTGCC |
| 15 | *evm.TU.ctg8474.31* | GTTTTCTGAAAGATTGGGGG | CAACTTCTGGCTAACACTCC |

**Supplementary Table 3** Quality Assessment of Transcriptome Sequencing Data for *T. ambiguum* under Cold Stress.

| **SampleID** | **ReadSum** | **BaseSum** | **GC(%)** | **N(%)** | **Q20(%)** | **Q30(%)** |
| --- | --- | --- | --- | --- | --- | --- |
| CK1 | 20746290 | 6204824065 | 41.51 | 0.01 | 99.33 | 97.58 |
| CK2 | 20231514 | 6052561578 | 41.59 | 0.01 | 99.33 | 97.63 |
| CK3 | 20930728 | 6256318163 | 41.75 | 0.01 | 99.34 | 97.67 |
| H2-1 | 20771380 | 6215570622 | 41.42 | 0.01 | 99.11 | 96.92 |
| H2-2 | 22757057 | 6814439431 | 41.28 | 0.01 | 99.16 | 97.06 |
| H2-3 | 23764240 | 7112116922 | 41.28 | 0.01 | 99.12 | 96.98 |
| H6-1 | 20440499 | 6105161806 | 41.62 | 0.01 | 99.41 | 97.91 |
| H6-2 | 21391950 | 6401109360 | 41.36 | 0.01 | 99.22 | 97.26 |
| H6-3 | 22113866 | 6619241828 | 41.25 | 0.01 | 99.16 | 97.31 |
| H12-1 | 20654180 | 6177963064 | 41.26 | 0.01 | 99.28 | 97.48 |
| H12-2 | 21054352 | 6298751520 | 41.3 | 0.01 | 99.34 | 97.63 |
| H12-3 | 20512721 | 6137774317 | 41.39 | 0.01 | 99.29 | 97.45 |
